# *Notes from the Field:* Firearm Homicide Rates, by Race and Ethnicity — United States, 2019–2022

**DOI:** 10.15585/mmwr.mm7242a4

**Published:** 2023-10-20

**Authors:** Scott R. Kegler, Thomas R. Simon, Steven A. Sumner

**Affiliations:** ^1^Division of Injury Prevention, National Center for Injury Prevention and Control, CDC; ^2^Division of Violence Prevention, National Center for Injury Prevention and Control, CDC.

The rate of firearm homicide in the United States rose sharply from 2019 through 2020, reaching a level not seen in more than 2 decades, with ongoing and widening racial and ethnic disparities (*1*). During 2020–2021, the rate increased again (*2*). This report provides provisional firearm homicide data for 2022, stratified by race and ethnicity, presented both annually and by month (or quarter) to document subannual changes.

## Investigation and Outcomes

National Vital Statistics System final mortality data for 2019–2021 and provisional mortality data for 2022, stratified by race and ethnicity, were downloaded via CDC WONDER.* Data are monthly for all groups except non-Hispanic American Indian or Alaska Native (AI/AN) and non-Hispanic Asian or Pacific Islander (A/PI) groups, which are presented quarterly because of small monthly counts. Corresponding population estimates were downloaded from the U.S. Census Bureau.^†^ Annual and monthly (or quarterly for AI/AN and A/PI) crude rates were calculated by race and ethnicity, with all rates expressed per 100,000 person-years. This activity was reviewed by CDC, deemed not research, and conducted consistent with applicable federal law and CDC policy.^§^

During 2022, the national firearm homicide rate decreased for the first time since the sharp increase from 2019 to 2020. Nonetheless, the rate in 2022 (5.9 per 100,000) remained substantially higher than the 2019 rate (4.4), corresponding to 5,223 more firearm homicides in 2022 than in 2019 (Table). Rate patterns show notable differences by race and ethnicity (Supplementary Figure; https://stacks.cdc.gov/view/cdc/133535). Rates among non-Hispanic Black or African American (Black), AI/AN, and Hispanic or Latino (Hispanic) persons were notably higher during the period from 2020 through 2022 compared with 2019. The annual rate among Black persons during 2022 (27.5) was lower than that in 2021 (30.4) or 2020 (28.3) but was still substantially higher than in 2019 (20.5). Among AI/AN persons, the rate during 2022 (9.3) exceeded the rates in both 2021 (7.7) and 2020 (7.9). During 2022, the rate among Hispanic persons leveled off (5.5) but remained higher than that in 2019 (3.8). Rates among non-Hispanic White and A/PI persons, although lower, also increased from 2019 to 2021, followed by a decrease in 2022.

**TABLE:** Firearm homicide annual rates and counts, by race and ethnicity — United States, 2019–2022

Race and ethnicity*	Rate^†^ (no.)			
	2019	2020	2021	2022
A/PI, NH	1.0 (202)	1.0 (208)	1.2 (241)	1.1 (233)
AI/AN, NH	6.4 (154)	7.9 (191)	7.7 (185)	9.3 (224)
Black or African American, NH	20.5 (8,438)	28.3 (11,832)	30.4 (12,721)	27.5 (11,565)
White, NH	1.6 (3,129)	2.0 (3,969)	2.1 (4,064)	2.0 (3,828)
Hispanic or Latino, any race	3.8 (2,301)	4.8 (2,947)	5.5 (3,455)	5.5 (3,500)
**Overall^§^**	**4.4 (14,414)**	**5.8 (19,384)**	**6.3 (20,958)**	**5.9 (19,637)**

**Sources:** CDC WONDER; U.S. Census Bureau (NC-EST2020-ALLDATA; NC-EST2022-ALLDATA).

**Abbreviations:** A/PI = Asian or Pacific Islander; AI/AN = American Indian or Alaska Native; NH = non-Hispanic.

* Persons within some racial and ethnic groups, particularly AI/AN persons, might be undercounted because of misclassification. https://www.cdc.gov/nchs/data/series/sr_02/sr02_172.pdf; https://www.cdc.gov/nchs/data/nvsr/nvsr70/NVSR70-12.pdf

^†^ Crude rates represent the number of firearm homicides per 100,000 persons.

^§^ Rates and numbers for the “Overall” category include the non-Hispanic multiple-race population grouping, not shown separately in the table.

## Preliminary Conclusions and Analysis

Although the overall national rate of firearm homicide decreased from 2021 to 2022, the rate remained higher than in 2019. The onset of higher rates has been attributed to a range of factors, including economic and social stressors and disruptions in health and emergency services related to longstanding systemic inequities (such as employment or housing), which were worsened by the COVID-19 pandemic (*1*,*3*,*4*).

Although firearm homicide rates decreased for some groups in 2022, rates remained elevated overall compared with 2019, particularly among Black persons, and the rate increased among AI/AN persons. Continued prevention efforts, particularly those addressing social and structural conditions that contribute to violence, are needed to mitigate risks and inequities. These efforts include policies and programs promoting economic and housing security, hospital and community-based outreach and violence interruption programs, initiatives to enhance secure firearm storage to prevent unauthorized access or use, environmental changes such as remediating vacant lots, and therapeutic approaches to address trauma.^¶^ Such efforts can be furthered through partnerships involving community organizations that serve and represent those most affected by violence.

## Footnotes

* https://wonder.cdc.gov (Accessed August 10, 2023). Persons within some racial and ethnic groups, particularly AI/AN persons, might be undercounted because of misclassification. https://www.cdc.gov/nchs/data/series/sr_02/sr02_172.pdf; https://www.cdc.gov/nchs/data/nvsr/nvsr70/NVSR70-12.pdf

† Monthly population estimates, by age, sex, race, and Hispanic origin, April 1, 2010–July 1, 2020 (NC-EST2020-ALLDATA to cover January 2019–March 2020) and monthly population estimates by age, sex, race, and Hispanic origin, April 1, 2020–July 1, 2022, with short-term projections to December 2023 (NC-EST2022-ALLDATA to cover April 2020–December 2022). https://www.census.gov (Accessed June 29, 2023).

§ 45 C.F.R. part 46, 21 C.F.R. part 56; 42 U.S.C. Sect. 241(d); 5 U.S.C. Sect. 552a; 44 U.S.C. Sect. 3501 et seq.

¶
https://www.cdc.gov/violenceprevention/firearms/fastfact.html
